# ‘Bending’ against straightening devices: queer lived experiences of sexuality and sexual health in Bangladesh

**DOI:** 10.1186/s12889-023-15085-0

**Published:** 2023-01-25

**Authors:** Prima Alam, Cicely Marston

**Affiliations:** Department of Public Health, Environments and Society, Faculty of Public Health and Policy, London School of Hygiene and Tropical Medicine, London, UK

**Keywords:** Sexuality, Young people, Bangladesh, Heteronormativity, Qualitative research

## Abstract

**Background:**

Despite global data around increased health risks among sexual and gender diverse populations, lived experiences of young lesbian, gay, bisexual, transgender, queer or questioning, and others (LGBTQ+) people are often ignored in mainstream health research. This is particularly evident in countries such as Bangladesh where the rights of sexual minorities are not recognised. This article looks at queer lived experiences of sexuality and sexual health within such a context. We use the phenomenological framework of heteronormative ‘straightening devices’ – mechanisms working to direct people towards heterosexuality, gender conformity, and procreative marriage – to identify ‘invisible’ structures upholding normative sexual behaviours and see how young people in Bangladesh navigate these in their everyday lives.

**Methods:**

This article is based on qualitative data collected in Dhaka, Bangladesh over nine months in 2019 as part of the first author’s doctoral research. Using thematic analysis, we draw on experiences of normative sexual expectations from biographical in-depth interviews with 14 purposively sampled LGBTQ + individuals aged 18 to 24.

**Results:**

Respondents identified heteronormative expectations around gender norms of traditional behaviour and presentation for men and women as well as parental expectations of compulsory heterosexuality through marriage. These straightening devices existed at multiple levels, including individual, interpersonal, community, and societal. The four main themes around straightening devices include marriage norms for women; harassment of feminine-presenting bodies in public spaces; heteronormative healthcare; and consequences of not embodying heteronormativity.

**Conclusion:**

Our study highlighted young people’s everyday experiences of having to ‘bend’ to – and against – heteronormative straightening devices at home, in public spaces, and within institutions such as healthcare in Bangladesh. The exploration of queer experiences provides new insights into context-specific ways in which sexual and gender diverse people understand themselves. Further research using the framework of straightening devices can help public health professionals to identify more ‘barriers’ confronted by sexual and gender diverse young people.

## Background

Health needs and experiences of sexual and gender diverse populations – such as lesbian, gay, bisexual, transgender, queer or questioning, and others (LGBTQ+) individuals – are often overlooked within healthcare settings or assumed to be indistinguishable from that of their heterosexual and/or cisgender-conforming contemporaries [1–4]. However, global data reveal increased risk of poor mental health, discrimination, violence, victimisation, and higher rates of suicide as well as substance use among young LGBTQ + populations [4–8]. LGBTQ + individuals also face specific challenges related to their identity during adolescence – such as internalising negative societal messages of gender and sex as part of their self-image – and they could benefit from the support of health professionals to tackle them [9–11]. Consequently, health disparities persist and reflect larger sociostructural inequities which negatively impact the health of LGBTQ + young people around the world [2, 3, 9, 12, 13].

Despite statistics around ‘increased health risks’ due to discrimination and bullying, there is very little research investigating LGBTQ + young people’s views and lived experiences of sexual behaviours [5]. Lived experiences of sexual and gender diverse young people have been largely ignored in mainstream health research and policy, particularly in countries where the rights of sexual and gender diversity are not recognised [1, 2, 8, 11]. There is a scarcity of research on the sexual practices, lived experiences, and health concerns of LGBTQ + youth in South Asia where heteronormative patriarchy continues to be a dominant paradigm [11, 14–18]. For example, Bowling and colleagues [8] argue that the criminalisation of same-sex sexuality in India had ‘laid the foundation for ostracisation, sanctioned stigma and furthered injustices for many’ by forcing individuals to hide their sexuality. However, research in the area of LGBTQ + health in South Asia has disproportionately focused on HIV risk and prevention [8, 19–22]. This absence of diverse narratives around sexuality further perpetuates prejudices against LGBTQ + people and may negatively influence their access to high-quality healthcare.

### Heteronormativity in Bangladesh

As sexual diversity in Bangladesh ‘is not acknowledged socially or legally’, there is a lack of research around the sexual lives and health of LGBTQ + young people [14]. Unlike neighbouring Nepal and India, Bangladesh continues to criminalise consensual same-sex sexual conduct under Sect. 377 of its penal code as introduced by British colonial rule. Discrimination and violence against sexual and gender diverse individuals in Bangladesh has been described as ‘pervasive and ongoing’ [23]. The brutal murders of prominent LGBTQ + activists Xulhaz Mannan and Mahbub Rabbi Tonoy in April 2016 has made the situation even more dangerous for Bangladesh’s LGBTQ + community [24, 25]. Since the incident, many LGBTQ + people have either left the country or gone ‘underground’ due to safety concerns [24].

As with other postcolonial nations in the region, procreative heterosexual marriage is central to the regulation of sexuality in Bangladesh and provides a dominant reference for sexual morality [26]. Multiple sexual expressions are ‘sanctioned or tolerated or ignored, as long as such activities remain hidden from the public gaze’ and do not disrupt the ideal of procreative heterosexual marriage [26]. Heteronormativity – framed under strict gender binaries, compulsory heterosexuality and marriage normativity – has become inscribed into postcolonial nations’ legal frameworks and gradually established as universal; ultimately negating the South Asian’s own history of sexual diversity [27–29].

This article explores the role of heteronormativity in shaping LGBTQ + identifying young people’s experiences of sexuality and sexual health in Dhaka, Bangladesh. We use the framework of heteronormative straightening devices to identify ‘invisible’ structures upholding normative sexual behaviours and see how young people in Bangladesh navigate these in their everyday lives. In particular, we ask: (1) What are the main heteronormative ‘straightening devices’ encountered by LGBTQ + young people? (2) How do LGBTQ + young people experience and navigate these straightening devices in their everyday lives? (3) What are the common health consequences of confronting these straightening devices?

Our analysis of empirical data – from the first author’s doctoral research – provides an opportunity to address the gap of sexual health research around sexual and gender diverse populations in Bangladesh. It also enables us to find out whether this framework is applicable within this context as we have little information from existing studies to assess this.

### Heteronormative straightening devices framework

Using the concept of orientation within phenomenology, Sara Ahmed [30] develops a compelling argument around how ‘heteronormative lines’ – lines upholding compulsory heterosexuality, gender conformity, procreative marriage, and nuclear families – direct us in certain ways throughout our lives and come to be seen as neutral starting points from which we experience the world. In other words, society assumes heterosexuality as the norm for all human experience and that everyone will attain heterosexual milestones – such as procreative marriage. This is further reinforced across all levels from the individual and inter-personal to the societal level.

‘Straightening devices’ are described as the mechanisms enforced to keep individuals aligned to heteronormative lines and ensure individuals do not deviate and become ‘wonky’ or ‘queer’ [30]. For example, the heterosexualisation of public space is naturalised by repetition of different forms of heterosexual conduct – such as heterosexual intimacy or nuclear families on billboards – and the passing by of some bodies and not others [30].

Ahmed [30] also notes that life courses follow a sequence which is also a matter of being directed along heteronormative lines: birth, childhood, adolescence, marriage, reproduction, and death. For a life to count as ‘decent’ it must take on the direction promised as a social good, which means reaching certain points along a life course. In such a case, a queer life within straight culture might be one that does not make such gestures of return – by not adhering to procreative marriage, for instance. This deviance is narrated as a loss of the possibility of happiness within queer lives and, from a lifeworld perspective, can offer insight into meanings of wellbeing [30].

## Methods

The findings for this paper are based on qualitative data the first author collected in Dhaka, Bangladesh over nine months from February to October in 2019 as part of their doctoral research. CM guided the research as PA’s primary supervisor. We predominantly draw on themes of heteronormativity from biographical interviews with LGBTQ + identifying university students and young professionals aged 18 to 24.

PA used purposive sampling to recruit 46 young people (aged 18 to 24) of varying social orientations – gender and sexual identity, religion, educational background, occupation, relationship status – based in Dhaka. These social orientations were selected based on the focus of the study and gaps identified through literature review (e.g. the lack of research focus on sexual and gender diversity, unmarried young people, and young people ‘transitioning to adulthood’) and communication with sexual health researchers in Bangladesh as part of the first author’s doctoral research [31–35]. These different social orientations also facilitated exploring a more diverse range of issues and meanings around sexuality [36–38].

The recruitment strategy was open and flexible in order to include a diverse range of experiences. Having previously worked in Bangladesh, PA initially approached potential participants through a range of academic and personal networks – including local queer advocacy groups and individuals. Local research assistants working with PA were also asked to utilise their networks to identify suitable individuals who may be interested in participating in the research. Using such referrals through networks assisted in building trust with participants. The research assistants themselves were all in their mid-20s and provided invaluable insights as locals growing up in Dhaka. They also assisted in discussing colloquialism and cultural references that the first author was less familiar with having been out of the country during most of their adolescence.

Participants included students at public universities, recent graduates, and young people in full- or part-time employment (both private and informal: such as teachers, garment factory employees, shop assistants etc.). Of the 46 total interviewees, 14 self-identified as LGBTQ+. Analysis of all interviews is included elsewhere in the first author’s thesis.

In-depth one-to-one biographic interviews comprised open-ended questions around lived experiences of gender and sexuality as well as life history. The first author conducted interviews in Bangla and began each interview with general conversation to make the interviewees more comfortable. All interviews were audio recorded. The interviews took between one and two and a half hours. Interview venues depended on where the respondents lived and were comfortable speaking, such as quiet cafes or private office space. All respondents provided written informed consent for participation in the research and were provided with details of local sexual and reproductive health services.

Four research assistants transcribed interviews in Bangla using a verbatim transcription protocol prepared by PA. Professional translators then translated these transcripts to English. Both Bangla and English transcripts were reviewed for quality and accuracy by PA and research assistants. PA used a phenomenological lifeworld approach to explore heteronormative straightening devices as identified and discussed in biographic interviews with LGBTQ + respondents in Bangladesh. The lifeworld approach can be used to explore how the ‘experiential side’ of health has a particular meaning for the person and must be attended to in order to better understand wellbeing [39]. PA followed a thematic approach to analysis by independently identifying and synthesising most prominent and recurring themes around sexuality through line-by-line open coding [36, 40–42]. The first author iteratively coded the interview transcripts using NVivo software and used the open coding nodes to formulate a codebook which was used for secondary coding through in vivo and process codes [43]. Additionally, PA also coded around the five domains of the phenomenological lifeworld (intersubjectivity, temporality, embodiment, emotions, and space) – as outlined by K Dahlberg, H Dahlberg, N Drew and M Nyström [38] and M van Manen [41] – for more details around experiences. PA clustered and reviewed codes into common (sub)themes and then analysed how these related to heteronormative straightening devices. Additionally, the first author referred to the ‘Consolidated criteria for reporting qualitative research’ (COREQ) for their doctoral thesis.

Ethical approval was granted by the London School of Hygiene & Tropical Medicine, United Kingdom, and from North South University, Bangladesh. PA obtained informed written consent from all respondents and used pseudonyms to maintain anonymity. Recordings and documentation related to the research did not contain any identifiable data.

## Results

All participants were aged 18 to 24 and self-identified as coming from middle-class or working-class backgrounds. Most identified as Muslim, while some participants also identified as Christian and Hindu. The majority had, or were pursuing, university education. Most participants lived with parents, on campus, or on their own at the time of the interviews. Table 1 provides a summary of sociodemographic characteristics of the 14 participants.

**Table 1:** Summary of participants’ sociodemographic characteristics

Characteristic	Category	Number of participants
Age	18–21	6
	22–24	8
Gender identity	Cisgender man	5
	Cisgender woman	5
	Transgender/non-binary	4
Sexual orientation	Heterosexual	2
	Gay/lesbian	8
	Bisexual/pansexual	4
Relationship status	Single	9
	In a relationship	5
Religious background	Muslim	9
	Hindu	3
	Christian/Buddhist	2
Occupation	University student	6
	Full-time employment	6
	Informal/part-time employment	2
Residence	Living with parents/family	5
	Living in rented accommodation	5
	Living in halls of residence/on campus	4
Socioeconomic background	Working class/lower income	6
	Middle class/middle income	8

Heteronormative expectations were apparent in participants’ narratives around gender norms of traditional masculine/feminine behaviour and presentation for men and women as well as compulsory heterosexuality through marriage (as expected by parents). Findings suggest that straightening devices operate at multiple levels, including individual, interpersonal, community, and societal. The four main themes around ‘straightening devices’ discussed in this paper include: marriage normativity for women; heteronormativity in public space; heteronormativity in healthcare; and consequences of not embodying heteronormativity.

### Straightening the life course: marriage normativity

Participants brought up family expectations around heterosexual marriage and suggested that most women in Bangladesh faced pressure from their families to get married. Amina – a 21-year-old lesbian woman living with her family in Dhanmondi, Dhaka – described expectations of being married by a certain age as commonplace for women:

Most go through the same norms that say, ‘you are a girl and you have to live like this.’ One of the rules is that since you are a girl, you have to get married. This is because of age. After a certain age, girls are more pressurised for marriage. (Amina, 21-year-old lesbian woman)

Amina was able to avoid these arranged marriages for a while, until “it all started again” as her grandmother’s death and her father’s ill health resulted in renewed pressure for her to get married.

While Amina felt she could not tell her family the truth about her sexuality, she had told them that she was not ready to get married. Despite this, her mother and brother kept ‘emotionally blackmailing’ Amina to get married to avoid being judged and disowned by their extended family.

As the ‘mental torture’ persisted, Amina became very distraught to the point that she was contemplating suicide. Amina decided to leave home as she felt she could not negotiate any further with her parents: “Every day of those weeks I spent thinking about suicide. I couldn’t take it anymore. I just wanted to escape.”

After about a week, Amina ran out of what little money she had and went to stay with her girlfriend. At this point Amina got in touch with her extended family and they assured her that if she came back home no one would pressure her to get married: “They told me, ‘Just come home. Your mother and brother won’t do anything to you.’ So, I decided to go back home based on their word.”

Amina explained how many other queer women in her friend group were also confronting similar pressures to get married, and that not all of them are able to navigate these pressures as she had: “There is a girl I know, she’s in a dire situation at home. There are a few in similar situations. Another person has been kicked out by her family. They are living on their own now.”

For Badol – a 20-year-old transgender man who was assigned female at birth and previously identified as a lesbian – marriage was used more directly as a straightening device. Unlike Amina, who did not disclose her sexuality to her family, Badol initially came out to his parents as queer. In response, Badol’s father arranged to get him married:

…My father pushed me to get married…as he knew about me and Noor [Badol’s girlfriend at the time]. And he fixed up a guy for me and pushed me into that marriage. (Badol, 20-year-old transgender man)

Badol found out about the marriage a week before the wedding ceremony and was convinced to make a promise to his father that he would go through with the marriage. From the start of their marriage, Badol’s husband was physically and emotionally abusive. Badol described feeling like a prisoner while enduring the ‘living hell’ of mental and sexual violence for two months:

I was being mentally tortured every moment and I felt like I was in prison. …He forced me from the first day of marriage. …At every moment I did not feel like myself. I mean, it felt like marital rape. (Badol, 20-year-old transgender man)

Despite being isolated from his family, Badol had “faith that Allah would find a way out for me”. He was finally able to leave the home of his abusive husband and file for divorce, with the help of his father.

After returning home, Badol continued to be open with his parents about his sexuality – at the time, he identified as a lesbian woman – but was told that his sexuality was unacceptable and that he would be ostracised in society. Badol faced further violence from his father in an attempt to ‘straighten’ him: “I fought a lot with my parent and my family, they even beat me for this [Badol’s sexuality]. My father beat me. He even arranged for goons to beat me up.”

### Performing straightness: heterosexualisation of public space

Outside of the home, straightening devices also became visible for the majority of queer respondents navigating public spaces. In particular, three queer participants shared in depth their experiences of public harassment and trauma when navigating public spaces.

Shayan – a 24-year-old dance instructor who identifies as a gay crossdresser – narrated numerous incidents of harassment by men throughout his life for as long as he could remember. For him, harassment was an everyday concern. Shayan generally tried to ignore verbal taunting as he was worried that the situation might escalate: “As long as they don’t come near me, as long as they don’t create problems, I will not say another word. Because it is when you try to talk to them, that you will have problems.”

Like Shayan, Trishna – a transgender woman working in the development sector – also used to stay quiet and ignore such encounters in an attempt to avoid escalating the harassment while growing up: “Whenever I tried to speak people would just mock me. They would make fun of me. I mean, I always felt uncomfortable.”

Non-binary student, Auvi, spoke of public harassment and emotional trauma as a wider issue not only for ‘queer-presenting’ people but also for women navigating public spaces because “people are going to be like dicks to you”.

Moreover, Auvi shared their past experiences of being able to ‘blend in’ when passing as man as well as being misgendered and seen as a woman in public spaces: “When you look like a woman, you get attention regardless of how you look. That’s something that’s been very common. Every single time I was either made to feel like a little girl or a woman in some way.”

It was apparent that persistent harassment affected the respondents’ emotional wellbeing. For instance, Shayan would remember various incidents – such as men on the streets making comments on Shayan’s way home – from earlier in the day after returning home and this would ‘ruin’ his mood.

Shayan was not always able to ignore derogatory comments, however. He recalled a recent experience when speaking back to a harasser, commenting on wanting to ‘chop off’ Shayan’s long hair, led to physical violence:

I was walking on the road, and a boy said to me, ‘Look at him! When I see him, I feel like yanking a fist full of his hair and chopping it off!’ He said something like that. So it was really irritating for me. Why should anyone, you know, talk to me like this? So I just turned around and said, ‘What is your problem?’ He replied, ‘So you talk!’ He said that, and then he grabbed hold of me and threw me on the road. …So then I said, ‘Why did you raise your hand to me?’ And he responded really harshly, ‘What are you going to do about it?’ (Shayan, 24-year-old gay man).

After arguing for a while longer, Shayan called his cousin for help and the cousin was able to intimidate the man who backed off immediately. However, the confrontation left Shayan deeply upset: “For a few days I became completely *abnormal* [depressed]. My hair was perhaps slightly longer than normal. I don’t see why that should be such a problem for us. I felt really bad.”

Trishna, who was assigned male at birth, also described the emotional impact of being questioned throughout her life – not only in public spaces but also by her family and peers – because she did not adhere to masculine gender norms:

I was being verbally abused with bad language and bad signs because I - my behaviour and attitude was feminine. My manner of speaking was feminine. Why did I behave more like a girl than a boy? Why was I like that? There were a lot of ‘whys’. It disturbed me a lot. (Trishna, 24-year-old transgender woman)

Auvi mentioned the emotional impact of having to negotiate their gender expression in public spaces by conforming to gender norms or presenting as queer:

I tried covering up, made me feel worse. I tried dressing more like myself. I got more attention… Started being like, ‘I don’t give a fuck about this shit because it’s too hot to wear an *orna* [scarf] and to have long hair and to wear full pants [trousers] and I’ll just deal with the fact that people are looking at me.’ (Auvi, 22-year-old non-binary person)

As a result, Auvi limited themself in terms of where they went and with whom. They avoided using public transport and going out alone, instead hanging out at friends’ homes where they felt safer.

Shayan, who lives by himself in a relatively low-income neighbourhood, mentioned that facing street harassment was unavoidable for him. Although Shayan tried to keep a ‘sense of courage’, he reported feeling particularly ‘insecure’ and ‘tense’ because he does not live in an affluent area of Dhaka:

I have to tolerate it [harassment in public spaces]. …It is not possible for me to live in a place like Gulshan or Banani. …So in local areas, these things are bound to happen.

… I do feel that sense of insecurity – about who is nearby, and who among them might create a scene. At times like that, I feel very [insecure]. (Shayan, 24-year-old gay man)

At the time of the interview, Auvi was about to go to Europe to do their master’s degree. For them, this presented an opportunity to be as queer as they wanted without the same sort of restrictions as they faced in Dhaka: “I imagine that a lot of this will change and not necessarily that I’ll become more sexual, but also just being able to present myself as somebody who doesn’t necessarily need to act in a particular way.”

It was notable that the respondents who reported being harassed thought they were harassed because they were seen to be presenting as feminine in public. Two queer-identifying participants, Farhana and Minhaj, both alluded to this as a product of patriarchy and gender expectations whereby women presenting as masculine were considered to be ‘upgrading’ and thus applauded, while men presenting as feminine were considered to be ‘downgrading’ and stigmatised:

“A girl wearing a shirt or suit is considered a ‘brave girl’ but a man wearing a skirt would be stigmatised… Patriarchy works both ways.” (Minhaj, 20-year-old gay man)

“When women are more like men it is considered ‘upgrading’ but [when] men are more like women then it is considered ‘downgrading’.” (Farhana, 23-year-old lesbian woman)

### Straightening as ‘care’: heteronormativity within healthcare

Healthcare was an important institutional straightening device as discussed by queer participants. In particular, transgender and gay participants spoke of concerns around, and experiences of, being discriminated against by healthcare professionals. In terms of perceptions, queer participants were of the view that disclosing their sexuality/being ‘found’ to be queer could result in discrimination from most healthcare providers. Most queer respondents, therefore, preferred to seek out LGBTQ + friendly doctors for sexual health issues.

For example, James – a 23-year-old gay master’s student – acknowledged that he wanted to have frequent three-monthly sexual health check-ups but faced difficulties in finding LGBTQ + friendly facilities:

I am facing this problem with regard to having the STD check-up [sexually transmitted infection testing] done. I have been unable to find a place in Bangladesh where I can go and have the check-up done. …The thing is, I had an STD check-up done quite a while ago. It has been almost a year, and the thing is, I have been wanting to have the check-up done once again. I think the check-up should be done quite frequently. Like, with a gap of about three months. (James, 23-year-old gay man)

For James, being tested was an important part of the process of negotiating safer sex – or deciding to have sex without a condom – when ‘hooking up’ with sexual partners:

Obviously, I would of course prefer safe[r] sex. And once things are finalised, obviously it is a good idea to have an STD check, in my opinion. Just to be sure. Then after having a discussion about it – what I mean is, after having a discussion, we can come to a decision, about whether you prefer safe[r] sex or not. (James, 23-year-old gay man)

Previously, James had used a confidential referral service organised by Roopbaan – a non-profit LGBTQ + platform – for sexually transmitted infection (STI) testing. However, increasing safety concerns for the LGBTQ + community following the 2016 murders of Roopbaan’s founder, Xulhaz Mannan – as well as fellow activist Mahbub Rabbi Tonoy – meant that such events had been discontinued: “Now everything has become totally challenging. I think that there are many security concerns.”

While the respondent did not share any experiences of discrimination at health facilities, he felt unsure about how health professionals would react to him requesting STI tests and did not want to “take that risk”.

Although James had not been able to find a workaround to the absence of appropriate sexual health services available to him, he had approached queer allies to advocate for this: “I have approached some of the doctors I know who are friends of the [queer] community. They have told me that they will try.”

The participant also pointed out that this was part of a wider sexual health issue, and that testing should be implemented by the government and made available to the whole population as STIs were a concern for everyone regardless of their sexuality.

While James and others spoke of their perceptions, three transgender respondents reported actually having negative experiences of being dismissed or misdiagnosed by psychiatrists. While Trishna – a 24-year-old transgender woman living on her own near Tejgaon, Dhaka – had sought out a healthcare provider for herself, transgender respondents Badol and Ria’s families had arranged for them to see psychiatrists as an intervention. Badol – who was assigned female at birth – explained that his parents thought he had ‘gone mad’ after he escaped an abusive marriage (arranged by Badol’s father in an attempt to steer Badol away from being in a lesbian relationship):

Sometimes I used to joke with my mum that she was doing all the household work alone, what if I brought a bride to help her? …They thought I was becoming more and more mad by the day.

[They thought that] I had gone through a lot in my life and had been hurt. Or a virus had attacked me. When I told my father about my relationship and Joba [Badol’s girlfriend at the time], he told me that this is not right and took me to a psychiatrist. (Badol, 20-year-old transgender man)

Both Badol and Trishna mentioned being misdiagnosed by psychiatrists who treated their sexual and gender identity as a mental illness:

The psychiatrist prescribed me lots of drugs. … I wasn’t able to function properly after taking those medicines. He also said that this [sexual identity] is a hallucination and a mental disorder. (Badol, 20-year-old transgender man)

They [psychiatrists] were treating it as some kind of a disease. They were thinking in that manner. I suffered from the side effects of wrongly prescribed medicine. I was prescribed medicine used to treat to people with psychosocial disorder. (Trishna, 24-year-old transgender woman)

For 19-year-old transgender student Ria, her first interaction with a counsellor – recommended to Ria’s sister through a family-friend – left her feeling dismissed and not heard when speaking about wanting to transition:

The counsellor asked me, ‘What would you even do in life as a woman?’ What does that mean?! I found that question so odd. You could ask, ‘What are your life plans as a woman?‘ But you can’t ask me what’s the point of being a woman or man. Am I meant to be thinking about the costs and benefits? (Ria, 19-year-old transgender woman)

After this negative interaction, a queer friend referred Ria to an LGBTQ-friendly psychiatrist. However, Ria was again disappointed as she was advised to ‘take more time’ before deciding to transition. In fact, the psychiatrist told her to come back when she was 25 years old: “They also said it [gender-affirming surgery] was illegal in Bangladesh. They said nothing is possible and told me to come see them again after I am 25.”

This left Ria even more frustrated as she expected the psychiatrist to be more understanding of her situation. She mentioned constantly struggling with her gender identity to the point where she ran away from home and attempted suicide twice. Ria felt like she was being asked to keep struggling and put her life on pause: “I was so hurt. …I would just start the [hormone] therapy at 25…then my life [as a woman] wouldn’t begin until after 30.”

In an attempt to convince his parents that he was not ‘going mad’, Badol took matters into his own hands and also made an appointment with a more sympathetic queer-friendly psychiatrist. In contrast to Ria’s experience, Badol was pleased with the appointment: “I fixed an appointment with him [a well-known psychiatrist]. When we met that psychiatrist, he told my father that this is completely normal, it’s natural and it’s not my fault.”

However, Badol’s father was ‘still in denial’ and not swayed by this second opinion because of concerns of social stigma due to Badol’s sexuality: “If we were living abroad then it would be fine with him. But, in Bangladesh, it is prohibited.”

Trishna sought the help of a psychiatrist once she had decided she wanted to transition. Although she could not recall the medication she was prescribed when she was misdiagnosed, Trishna remembered noticing multiple side effects – such as drowsiness, weakness, and back pain. These side effects not only adversely affected her efficiency and enjoyment of everyday activities at the time, but being on the medication also affected her ability to work as a performer:

I started to notice my body was weakening. I mean, I felt drowsy all the time. So, it was like - if I took the medicine at night, I kept on sleeping throughout the whole morning and woke up around 11or noon. I couldn’t keep my eyes open at all. I would eat something and then go back to sleep again. But that’s not like me at all. I’m a very hard worker. I enjoy working hard. My - that was not like my lifestyle. (Trishna, 24-year-old transgender woman)

The respondent decided on her own to discontinue the medication after about six months. One reason for this was because she felt she could not trust her psychiatrist as he did not listen to her concerns or make eye contact with her. Trishna summarised how distressing the whole ordeal was for her as she had expected better care from a medical professional:

…I tore up all my prescriptions and everything else. I spent two nights away from home. Two entire days. Because I felt something like that happened to me - OK so my friends did that to me, my family did that to me. They made fun of me. They didn’t treat me well. But how can a medical professional treat me the same? (Trishna, 24-year-old transgender woman)

As a result, she said she no longer trusts Bangladesh’s healthcare system and “can’t even trust to take paracetamols prescribed by Bangladeshi doctors”.

With the help of transgender friends in India, Trishna was able to begin hormone replacement therapy with a doctor in Kolkata, India:

He prescribed medicine only after doing all the tests. I completed a three-month course. I went to see him after. Then, he gave me more tests. He looked through my reports. I started my medication afterwards again. Then, what happened was that I started to change gradually. I mean, structural change. I am really well because of that, honestly. (Trishna, 24-year-old transgender woman)

Continuing the therapy was financially challenging for Trishna as she had to visit Kolkata every three months:

There are months when I have to be very careful with my money. Because I don’t have any extra income sources. I barely manage to save up and bear all my expenses with my salary. I mean, I am still struggling now. …I have to spend a lot. It’s a lot of money. (Trishna, 24-year-old transgender woman)

### Unbecoming straight: consequences of not embodying heteronormativity

Participants brought up multiple experiences of, and emotions around, not conforming to norms around heterosexuality and gender throughout their lives. In particular, respondents gave examples of confronting feeling ‘different’ from a young age and dealing with internalised homophobia as well as confusion over sexuality and gender identity through attraction to the same sex/gender dysphoria. Many described feeling ‘abnormal’, ‘unnatural’, ‘gross’, ‘dirty’, and ‘uncomfortable’ while exploring their own sexuality and gender identity.

Participants spoke about how they were identified and perceived to be different from their gender and sexuality conforming peers. For example, Shayan highlighted how not embodying heteronormative masculine traits in public was associated with being ‘found’ to be different and harassed as a result of this difference. Shayan mentioned that he had been persistently confronted by this type of harassment throughout his life, even when he himself did not understand what it meant:

I was perhaps five years old, or maybe six, and many people would call me ‘half ladies’. I didn’t even understand what that meant.

…You could see it in the way I moved, there were some biological issues. Those were things that they picked up on at that time. Or perhaps they just felt that there was ‘something a little effeminate about this one’. Because of that, they used to behave in this way with me. (Shayan, 24-year-old gay man)

Shayan, believed that it was something ‘genetic’ or ‘biological’ that made him identifiable as ‘different’ from men who conformed to masculine norms: “As people watch me, they realise that there is something about the way I move which perhaps makes them understand that there is something different about me. So, they say things [about me] like, ‘Hey, *bhabi* [sister-in-law] is here.’”

For some, gender dysphoria was felt more acutely during puberty. Ria and Trishna, two transgender women who were assigned male at birth, both described feeling emotionally troubled or restless and physically uncomfortable within their bodies throughout their childhood and teenage years. Ria described being restlessness about her gender from a very early age as well as feeling like she was trapped inside a male body: “[It is like] you’re one thing on the outside but you’re something else on the inside. …This feeling was coming from inside me. I was so restless. I couldn’t resolve the matter.”

Ria was in Year 7 when she first heard about gender reassignment and felt like she had to realise her ‘dream’ of becoming a woman by running away from home and becoming financially independently:

The first time I heard that it was possible to change to be a woman, my eyes were opened. That was my dream. …[I thought] now I have to solve my own problem. I felt like I had to get a job, had to save up money [for gender-affirming surgery] and be independent. (Ria, 19-year-old transgender woman)

Like Ria, Trishna recalled a similar feeling of constant discomfort within her body throughout her childhood and teenage years:

My body wasn’t responding the way a male body should. The male organs weren’t functioning the way they would do at that age. What was I supposed to do with an incompatible body? …Afterwards, when I realised that I couldn’t take it anymore - this went on until university. My change started after that. That’s when I took that decision [to transition]. (Trishna, 24-year-old transgender woman)

She went on to describe how she kept questioning whether she was ‘normal’ and ‘natural’. Finding answers to these ‘whys’ adversely affected her mental health and confidence, leading to a suicide attempt:

I went through a lot of mental trauma. I was- at a point I started questioning myself. Why am I like this? Why is this happening to me? That caused me to lose self-confidence and which caused me to one point attempt suicide. Just to find the answers to all the ‘whys’. Am I not a normal human being then? Am I really unnatural then? (Trishna, 24-year-old transgender woman)

Questions around being ‘normal’ came up in most interviews. There were assumptions that not embodying heterosexuality was associated with past sexual abuse and life challenges, contributing to feelings of guilt and confusion around sexual trauma. James described being sexually abused by a much-older male relative from the age of 10 to 13:

He [an older male relative], how can I put it, introduced me to kissing and other things like that. But I was not interested. Then again, what can I say, at such a young age, I did experiment with this new thing. And I also enjoyed what came out of my curiosity. (James, 23-year-old gay man)

As it was also around this age that James became more curious about his own sexuality, he remembered questioning whether the sexual abuse somehow shaped his sexuality and how people knew that he was ‘different’:

I don’t know how people can somehow understand…that I might be like this [gay]. If that was not the case, then I would not have been abused so many times. I was very confused at that time, wondering whether this was a problem with me. Wondering, ‘Is it OK? Is it natural?’ …Because of that, afterwards I suffered from guilt for a very long time - like, I was really confused. …[I thought,] ‘I am different, that’s why I got molested.’ (James, 23-year-old gay man)

Auvi also pointed out that they thought people generally perceived trauma as causing ‘deviance’ from normative behaviour, although for them it was the other way around: “I was like, ‘No, I was queer, that’s why I was given all this shit that caused the trauma.’…I think I was basically queer since I was born. I just didn’t have the words for it.”

Participants also spoke about difficulties in accepting their sexual identity. For example, James revealed that although he knew he was attracted to men, he felt ‘confused’ and was unable to accept this about himself for several years: “I was confused for a long time – I was unable to accept my own sexuality. For a long time, since 2009, I have known this about myself. But I was unable to accept it.”

Two bisexual women, Deepa and Zohra, recalled their first experience of same-sex attraction and described initially thinking it was ‘gross’, ‘dirty’, and ‘uncomfortable’. Both respondents acknowledged that these negative feelings were influenced by homophobia. They reconsidered the experiences on their own and started to be more accepting of their sexuality:

I started to have romantic thoughts about her [a friend from school], but I pushed those ideas away. Because I was kind of a homophobe. …My cousin was a lesbian but - I didn’t like this about her. I used to think of it as something dirty. So, when the same thing started to grow in me - I couldn’t accept it at first. I wasn’t scared of it, but I felt uncomfortable. I-I mean, I wanted to repress it. But I started to see that I can’t push these thoughts away anymore. I couldn’t stop thinking about her. And whenever I used to close my eyes, I had sexual thoughts about her. I would imagine things about her. Then I thought, ‘OK, fine. I should just accept it.’ (Deepa, 23-year-old bisexual woman)

The first time it happened I was like all-uh-I mean grossed out. I got grossed out by the experience. But, later on, I was like that- after I got grossed out, I was like- wait, no. If I was truly grossed out by it then it would be a bit different - I would have reacted a bit differently, right? (Zohra, 18-year-old bisexual woman)

While Deepa and Zohra’s narratives focussed on self-reflection around internalised homophobia in accepting their bisexuality, other participants mentioned finding support from peers, television shows, and the internet. Most queer young people identified LGBTQ + visibility and representation as a point when they began feeling ‘natural’, ‘normal’, and ‘less alone’ regarding their sexual and gender identities. Three participants gave examples of specific Hindi and English television shows which helped them to discover more about sexuality. For instance, Ria said that the first time she came across non-heterosexual representation as something ‘normal’ was while watching an Indian television show called ‘*Kaisi Yeh Yaariaan*’ (‘How is This Friendship’) which had a gay storyline. She specifically recalled one of the characters coming out to his mother as gay, which made Ria think it might be possible for her to confide in her own parents. Amina also mentioned feeling excited to see lesbian intimacy represented for the first time on an Indian show called ‘The Other Love Story’. She remembered really looking forward to the show: “Addheyian and Anchaal were the names of the two characters. I started watching that. I used to wait for new episodes every week.”

James remembered seeing his sexuality as something ‘natural’ and feeling very ‘supported’ when he first started watching British television show ‘Queer as Folk’:

There was a television series that really influenced me. The subject was learning to accept oneself. … ‘Queer as Folk’. I felt so supported after I watched that TV series. I liked it so much, that I thought, No, it’s quite natural. People can be like this. There is nothing wrong with that. (James, 23-year-old gay man)

Participants also spoke about forming friendships with others within the LGBTQ + community – both virtually and in person – while first learning about sexual and gender diversity. For example, Trishna said having online transgender friends made her feel more ‘relaxed’ and ‘less alone’. Trishna’s friendships also helped improve her mental health by giving her a sense of relief and stopping what she referred to as an ‘addiction’ to suicidal thoughts: “I made a lot of friends online. …It helped me to stop my suicidal *addiction*. …When I saw that I wasn’t alone. There are many people who are facing similar situations. This is normal. I felt relieved after that.”

Trishna fondly remembered how she was ‘inspired’ to transition by a transgender friend in India – a friend who sadly later took her own life. As well as inspiring Trishna, Asha also provided practical support by taking Trishna to see a doctor in Kolkata, India:

I had an internet friend called Asha. She is no more. She committed suicide. …She was a transgender woman. I was inspired by her. [I thought] she’s doing it, so why not me? Why am I not taking the step? She was my main inspiration behind my decision to change. She was the one who took me to the doctor. (Trishna, 24-year-old transgender woman)

## Discussion

Our findings around ‘straightening devices’ in Bangladesh support global literature about discrimination and inequalities confronted by LGBTQ + youth – across individual, interpersonal, community, and societal levels – and adverse consequences for their mental health and wellbeing [2, 3, 5, 8, 9, 44–46]. Concern around concealing sexual or gender identity as a matter of personal safety in public spaces and healthcare facilities resulted in strong feelings of discomfort among queer young people. Ahmed [30] described these emotions as a common feature for individuals who are perceived to not conform to heteronormative society. As a straightening device, queer people may be asked not to make others feel uncomfortable, by not displaying signs of queerness in heteronormative spaces [30]. In this manner, availability of comfort for normative bodies depends on the labour of sexual and gender diverse bodies through concealment. This burden of concealment and perceived ‘failure’ to embody heteronormativity resulted in feelings of guilt and shame for participants in our study. Experiences and expectations of concealment varied depending on factors such as ability to pass as cisgender and heterosexual. For example, as women routinely face street harassment due to widespread gender discrimination in South Asia [47–50], participants perceived that passing as masculine in public spaces would draw less attention.

Overall, perceived insensitivity towards gender and sexual diversity – in addition to social stigma of gender and sexual diversity and taboos around sexuality – left LGBTQ + young people without adequate support from institutions as well as from their families and wider community. Evidence from global research suggests that lack of supportive environments at home, in school and healthcare settings further compounds the emotional challenges of living outside of heteronormativity [46, 51–56]. Adverse health implications such as trauma, depression, anxiety, and suicidal thoughts as described by all participants in our studies are also widely reported in mainstream research on LGBTQ + experiences [2–5, 7–10, 13, 44–46]. In particular, transgender youth in our study reported being the most affected by heteronormativity in healthcare because they felt disrespected and stigmatised for their gender identity – the very reason they had sought help in the first place. Moreover, healthcare providers were perceived to have inadequate knowledge of gender reassignment and often misdiagnosed transgender patients. Transgender youth in high-income settings also reported having similar negative healthcare experiences [57–59].

Wider research recommends a range of interventions to reduce health disparities by improving social support for LGBTQ + youth – such as promoting family acceptance, providing safety nets for youth in case family relationships breakdown, and training health professionals [9, 51, 52]. Narratives of young people in our research indicate perceived sociostructural barriers to implementing such interventions. In this context, we as researchers and practitioners must ensure the same level of support in places where queer rights are not recognised by leveraging local sexual health programmes and expertise. It is important to identify those willing to support such initiatives and avenues of sexual health where rights-based progress is possible. Further research is needed to see how effective these initiatives could be at facilitating ‘bending’ against straightening devices in Bangladesh.

This study examined lived experiences of self-identifying LGBTQ + young people in Dhaka within the phenomenological framework of heteronormative straightening devices. Although some of the participants grew up in different areas of the country, most of the narratives referring to public spaces and health services were based on urban settings. As such, further research is needed to understand the experiences of peri-urban and rural areas where young people may be facing different challenges. Similarly, we were unable to recruit intersex people and their experiences should also be investigated in future work. From a phenomenological standpoint, it would be useful to explore each of the ‘straightening devices’ – as well as specific sexual and gender identities – in more depth to better understand the essence of lived experiences. Given that our research focus was lived experiences of young people, we could not address the perspective of other actors who may be upholding heteronormative straightening devices – such as healthcare professionals or parents of young people. Examining structural challenges may provide more context-specific information on how to effectively advocate for the rights of queer young people. Finally, due to the dearth of research on sexual and gender diverse young people in Bangladesh, we had to rely on reviewing global literature. As many of these other studies were based in high-income countries, the recommendations need to be further examined to be applicable in Bangladesh.

## Conclusion

Our study highlighted young people’s everyday experiences of having to ‘bend’ to – and against – heteronormative straightening devices at home, in public spaces, and within institutions such as healthcare in Bangladesh. Young people in our study reported having very little social support and navigating this by seeking representation in the media and friendships with other LGBTQ + identifying people. The research focus intended to move away from causes and treatment of ill health in favour of a contextualised people-centred approach to understanding wellbeing. In doing so, our data highlight an urgent need for more inclusive and holistic healthcare – with a focus on mental wellbeing and support – from a rights perspective. This is an important contribution as there is limited published in-depth qualitative research in the field of sexual health in Bangladesh. The exploration of queer experiences of heteronormativity provides new insights into crucial context-specific ways in which sexual and gender diverse people understand themselves [20, 26]. The study provided an opportunity to see whether heteronormative straightening devices were applicable within the context of Bangladesh as there is little information from existing studies to assess this. The health needs highlighted by participants – such as mental health consequences of persistent harassment – have implications for future research as well as interventions. Further research using the framework of straightening devices can help public health professionals to identify more ‘barriers’ confronted by sexual and gender diverse young people.

## Data Availability

The datasets generated and/or analysed during the current study are not publicly available as they contain sensitive information and data could potentially identify participants. Data are available from the corresponding author, Prima Alam, on reasonable request.

## List of abbreviations

LGBTQ+: Lesbian, gay, bisexual, transgender, queer or questioning, and others
STD: Sexually transmitted disease
STI: Sexually transmitted infection
